# Stevens Johnson Syndrome: Past, Present, and Future Directions Gynecologic Manifestations and Management in SJS/TEN

**DOI:** 10.3389/fmed.2022.874445

**Published:** 2022-07-04

**Authors:** Michelle A. DenAdel, Sarah E. Hendrickson, Esther Fuchs

**Affiliations:** Department of Obstetrics and Gynecology, University of Washington, Seattle, WA, United States

**Keywords:** Stevens Johnson Syndrome (SJS), Toxic Epidermal Necrolysis (TEN), vulvovaginal sequelae, gynecologic manifestations of SJS/TEN, treatment of vulva and vagina, standardized protocol for SJS/TEN

## Abstract

Stevens Johnson Syndrome (SJS) and Toxic Epidermal Necrolysis (TEN) are severe mucocutaneous hypersensitivity disorders characterized by sudden onset epidermal necrosis. Acute manifestations of SJS/TEN often include vulvovaginal erosions, ulcerations, vaginal discharge, bleeding, vaginal pain, dysuria, and urinary retention. If not treated, this can lead to complications such as vulvovaginal adhesions, vaginal stenosis or dryness, pain, dyspareunia, bleeding, and adenosis. Even with adequate treatment, there are lasting impacts including difficulty with vaginal exams and psychological distress. Early recognition and treatment of vulvovaginal involvement are crucial to preventing severe sequelae. Despite the potentially devastating consequences of genitourinary involvement of SJS/TEN, involvement of the mucocutaneous surfaces of the vulva and vagina is inconsistently documented, and protocols for treatment and follow-up are not well-established. The treatment of vulvovaginal involvement relies largely on expert opinion, and there is little data on the efficacy of suggested management. The goal of this review was to identify whether establishing a clinical pathway increased treatment of vulvovaginal SJS/TEN and to optimize our standardized protocol to prevent genitourinary sequelae.

**Methods:**

We conducted a retrospective chart review of female patients with SJS/TEN at Harborview Medical Center, University of Washington from 2008 to 2021. Demographic and clinical data including gynecologic consultation, exam findings, treatment regimens, and outpatient follow-up were collected from the electronic medical record. We compared data before and after implementation of a clinical care pathway in 2017.

**Results:**

We reviewed a total of 88 charts of women with possible SJS/TEN between 2008 and 2021. Of these 88 charts, 77 were found to have clear biopsy proven diagnosis of SJS/TEN. A total of 42 patients were found to have vulvovaginal involvement (55%) and gynecology was consulted in 43% of cases. 50% of patients (*n* = 21) with vulvovaginal involvement were recommended treatment with vaginal dilators and steroid ointment and 34% of patients with genital involvement received no treatment.

Between 2008 and May of 2017 (pre-protocol), we found 55 patients with SJS/TEN. 55% of patients (*n* = 29) had vulvovaginal involvement (*n* = 26 vulvar, *n* = 21 vaginal). Gynecology was only consulted in 26% (*n* = 14) of patients. Of the 21 females with vaginal involvement, only 38% (*n* = 8) had dilators/vaginal molds with steroid ointment recommended. Of the 26 females with vulvar involvement, 31% (*n* = 8) had no vulvar treatment recommendations with the remaining 69% having some documentation that ranged from gauze placement only (19%) to topical lidocaine, barrier cream, antibiotic or antifungal cream/ ointment, lubricant, or topical steroid ointment (50%). Menstrual suppression was recommended in 38% (*n* = 9) of menstruating females. An antifungal medication was only prescribed in 4% of patients.

Following implementation of the clinical pathway for the treatment of SJS/TEN in 2017, 22 females with SJS/TEN were identified. 72% (*n* = 16) had documented vulvovaginal involvement (*n* = 16 vulvar, *n* = 9 vaginal). Gynecology consultations took place in 86% (*n* = 19) of patients. We identified several improvements after implementation of the protocol. Gynecology consults overall increased from 26% pre-, to 86% post-protocol. For patients with vulvovaginal involvement, consultations were completed in 93% compared to 50% prior to protocol. Of note, the finding of vulvovaginal lesions increased from 53 to 72%. Dilator use with topical steroid ointment was consistently recommended, as was antifungal use and menstrual suppression.

**Conclusion:**

Having a protocol in place for treatment of female patients with SJS/TEN increased the consistency of Gynecologic consultation and the documentation and treatment of vulvovaginal SJS/TEN. We identified the need to improve clinical follow-up after discharge from the hospital, which could be arranged as multidisciplinary visits and would be a good option to assess long-term outcomes (pain, sexual activity, etc.). With regards to future directions, we are in the process of assessing long-term data on quality of life and sexual functioning. The impact of treatment in the acute setting on the development of chronic sequelae needs to be established, as does the management of long-term sequelae like vaginal dryness, pain, dyspareunia. The role of local estrogen and vaginal laser still needs to be explored. Pelvic floor physical therapy might play a significant role in rehabilitation and has yet to be studied.

## Introduction

Stevens Johnson Syndrome and Toxic Epidermal Necrolysis are severe mucocutaneous reactions characterized by sudden onset epidermal necrosis. SJS/TEN is more commonly seen in women than men and is most often triggered in response to a medication (1). Mucosal surface involvement is often widespread, involving the respiratory, gastrointestinal, and genitourinary tracts (2, 3). The reported prevalence of vulvovaginal involvement in patients hospitalized with SJS/TEN is extremely variable, was previously estimated to be as high as 70% (2, 4–6) and seems to be higher with routine consultation of a specialist in Gynecology. Failure to recognize and treat vulvovaginal SJS/TEN has the potential for severe acute and chronic morbidity (4), including vulvovaginal adhesions, vaginal stenosis, vaginal dryness, pain, dyspareunia, bleeding, adenosis, and psychological distress. Even when identified and treated, available data suggest that up to one third of patients develop chronic sequelae (2). Despite the potential for severe consequences, because of the focus on critical care in the acute phases of the illness and the sensitive nature of a gynecologic exam, it is possible that pelvic exams are being deferred, and vulvovaginal SJS/TEN is likely underrecognized.

Reported genitourinary symptoms frequently include pain, swelling, and dysuria (1). The importance of a comprehensive total body examination is well-documented, including the examination of the vulvar mucosa, perineum, perianal skin, and anus. Upon examination, acute vulvovaginal SJS/TEN most often present as erosions and ulcerations. Though a speculum exam is necessary for identification of vaginal lesions, experts have suggested assuming and treating possible vaginal involvement because of the painful and potentially distressing nature of such exams (2).

Treatment of mucosal involvement in the vulva or vagina relies largely on expert opinion, as there are no prospective trials to study its treatment. Current practice typically includes the use of topical application of corticosteroids, vaginal dilator therapy, menstrual suppression (1) and a Foley catheter with the goal of decreasing adhesion formation and agglutination, vulvar pain, and limiting metaplastic changes in affected tissue. Even when protocols exist at individual institutions, there is little data on the effectiveness of such protocols, or the degree to which they are followed (7).

Harborview Medical Center, University of Washington in Seattle, USA, is the only level one adult and pediatric trauma and burn center in Washington State and receives multistate referrals. In June of 2017, a Clinical Care Pathway for the treatment of patients with SJS/TEN was implemented. This pathway document is an institutional protocol for treating patients with SJS/TEN and provides recommendations for the evaluation and treatment of vulvovaginal involvement in SJS/TEN. It emphasizes consistent consultation of the Gynecology service with the goal of evaluating the patient and educating the patient and family regarding the treatment. The pathway document outlined several recommendations to aid the completion of a thorough gynecologic evaluation. This includes requesting notification of the Gynecology team prior to a procedure in the operating room to allow gynecological exam under anesthesia, including speculum examination to assess vaginal involvement. Treatment recommendations were protocolized. For all women or girls (who have been sexually active or who use tampons) with documented vaginal involvement, vaginal dilator therapy with concurrent use of steroid ointment was recommended to decrease risk of vaginal agglutination and adhesion formation. The protocol standardized vaginal dilator use to 20–30 min 2–3 times daily with generous application of the chosen steroid ointment (i.e., Betamethasone 0.05–0.1% or TAC 0.1% ointment). If unable to use steroid ointment, a water-based lubricant is acceptable. Ointments have fewer additives and are preferred to creams that can contain alcohol which causes a burning sensation on raw surfaces. It suggested consideration of menstrual suppression to decrease the risk of vaginal adenosis and for ease of hygiene. Vaginal antifungals were recommended to be used as needed for patients receiving long term antibiotics and to counteract the vaginal steroid which could promote fungal overgrowth. It advised that in the case of vulvar involvement only, vulvar mucous membranes should be treated similar to vaginal mucous membranes to decrease labial agglutination and scarring through application of topical steroids and manual separation of the labia two or three times daily with an impregnated gauze.

The goal of this study was to identify whether the protocol increased treatment of vulvovaginal SJS/TEN and to optimize our standardized protocol to prevent genitourinary sequelae.

## Methods

We conducted a retrospective chart review examining the frequency and treatment of vulvovaginal involvement in female patients with SJS/TEN before and after the implementation of a pathway for treating SJS/TEN at our level one burn center in the Northwest of the United States of America. We reviewed charts from 2008 to 2021 (14 years) and identified 77 patients with biopsy proven SJS/TEN (out of a total of 88 females with SJS/TEN). Inclusion criteria were a female patient admitted to the burn unit of our institution with SJS/TEN between 2008 and 2021. Vulvovaginal involvement was established by physical exam documentation. Treatment regimens were evaluated by reviewing a combination of the discharge summary, medication list, and gynecology consult note as well as other physician and nursing documentation. Post-hospitalization outpatient gynecologic follow-up from 2017 to 2021 was reviewed within our hospital system and using electronic access to other clinic and hospital systems. Descriptive statistics were reported as % (*n*) for all categorical variables and as a mean or count for all continuous variables. Fisher exact test with significance set at *p* < 0.05 was used to compare categorical variables pre and post implementation of treatment pathway.

Letters were sent to the patients with the goal of obtaining long term data on quality of life and sexual functioning following vulvovaginal sloughing due to TEN and SJS.

## Results

From 2008 to 2021, a total of 77 female patients admitted with biopsy proven SJS/TEN were identified. A summary of patient characteristics and treatment can be found in Table 1. The age of patients with SJS/TEN ranged from 6 to 93 years old with a mean age of ~45 years old. 55% of the 77 cases were classified as SJS and 43% were TEN. 55% of the patients with SJS/TEN had documented vulvar involvement (*n* = 42) and 39% had vaginal involvement (*n* = 30). A gynecology consult was obtained in 43% of patients with SJS/TEN. 49% of menstruating patients received menstrual suppression (*n* = 17), and 17% of patients were recommended an antifungal (*n* = 13).

**Table 1:** Demographics and clinical variables of patients with SJS/TEN from 2008 to 2021.

**Variable**	**% (mean or count) *n* = 77**
Age (years old)	6–93 (45)
**Menstrual status**
Premenstrual	4% (3)
Menstruating	45% (35)
post-menstrual/hysterectomy	43% (33)
**Sexual activity**
Yes	17% (13)
No	22% (17)
Not specified	63% (48)
**Diagnosis**
SJS	55% (42)
TEN	43% (33)
**Extent of vulvovaginal involvement**
Vulvar involvement	55% (42)
Vaginal involvement	39% (30)
Gynecology consult	43% (33)
Vaginal treatment recommendations (a)	70% (21)
Dilator recommended	70% (21)
Dilator used	67% (20)
Steroid cream/ointment	71% (30)
No vulvar treatment recommendations +	19% (8)
Vulvar treatment recommendations (b)	81% (34)
Gauze only	19% (5)
Topical Steroid	45% (19)
Other	36% (15)
Menstrual suppression (c)	49% (17)
Antifungal	17% (13)

*(a) recommendation for vaginal dilator of the patients with documented vaginal involvement (n = 30). (b) Vulvar treatment recommendations of the patients with vulvar involvement (n = 42). (c) menstrual suppression of menstruating patients (n = 35)*.

Prior to implementation of the clinical care pathway (2008–2017), 55 patients were identified with a clear biopsy proven diagnosis of SJS/TEN. The mean age was 45 years old (6–93). 47% of patients (*n* = 26) had documented vulvovaginal involvement, 21 of which also were found to have vaginal involvement. Gynecology was consulted in 26% (*n* = 14) patients. Of the 21 females with vaginal involvement, only 38% (*n* = 8) received treatment with dilators/vaginal molds with steroid ointment. Of the 26 females with vulvar involvement, 31% (*n* = 8) received no vulvovaginal treatment. The remaining 69% had some documented treatment that ranged from gauze placement only (19%) to topical lidocaine, barrier cream, antibiotic or antifungal cream/ointment, lubricant, or topical steroid ointment (50%). Menstrual suppression was recommended in 38% (*n* = 9) of menstruating females. An antifungal medication was prescribed for 4% of patients.

Letters were sent to that patient group with the goal of obtaining long term data on quality of life and sexual functioning following vulvovaginal sloughing due to TEN and SJS. However, of the 45 letters sent (out of 55 females, 9 deceased, one location not available), only 5 patients responded and therefore feedback about long-term sequelae was insufficient.

Charts from 2017 to 2021, following implementation of the standardized protocol, were also reviewed. 22 females with SJS/TEN, ages 7–83 with mean age 46 years, were included. Seventy-two percentage (*n* = 16) of patients had documented vulvovaginal involvement, vulvar 72% (*n* = 16) and vaginal 41% (*n* = 9). There was no difference in rates of vulvar involvement between patients with SJS vs. TEN (*n* = 8 for both groups). The rates of vaginal involvement were also not significantly different between patients with SJS vs. TEN (*n* = 5 and 4, respectively). Gynecology consultations took place in 86% (*n* = 19) of patients and only one patient with possible vulvovaginal involvement lacked a consult, while vulvovaginal involvement in 2 females was not documented. Two patients with a gynecology consult were recommended prophylactic vulvovaginal treatment, despite lack of vulvovaginal involvement at the time of examination. One patient was recommended to use a vaginal dilator with a steroid ointment, and the other patient was recommended topical steroid both internally and externally. Both patients were recommended antifungal treatment. Recommendation for post-hospitalization follow-up with gynecology was documented in 9 out of the 20 patients (45%) that were not deceased at the time of discharge, but only 2 females had completed a documented follow-up visit within 6 months.

We are currently in the process of contacting patients that were hospitalized after 2017, with the goal of obtaining long term data on quality of life and sexual functioning.

We found several improvements after implementation of the protocol (Table 2). Gynecology consults increased from 26% pre- to 86% post-protocol (Fisher exam test statistic value <0.00001 at *p* < 0.05). For patients with vulvovaginal involvement, consultations were done in 93% compared to only 50% prior to protocol (Fisher exam test statistic value <0.00001 at *p* < 0.05). Documentation of vulvar involvement increased from 47% pre-protocol to 72% post-implementation (Fisher exact test statistic value 0.048). Documented vaginal involvement remained largely unchanged, 40% pre-protocol to 41 % post-protocol. There was a significant increase in the use of vaginal dilators and steroid cream (Fisher exact test statistic value 0.0178) and in the use of an antifungal (Fisher exact test statistic value 0.0004 at *p* < 0.05). There were significant increases in treatment for patients with vulvar involvement and in the use of menstrual suppression (both *p* < 0.05). If a gynecology consult was omitted, rather no vulvovaginal exam than a negative exam was documented. Additionally, documentation of sexual activity and pregnancy status improved significantly.

**Table 2:** Demographics and clinical variables of patients with SJS/TEN.

**Variable**	**% (mean or count)**	**% (mean or count)**
	Total *n* = 55	Total *n* = 22
	(a) *n* = 21	(a) *n* = 9
	(b) *n* = 26	(b) *n* = 16
	(c) *n* = 24	(c) *n* = 11
	2008–2017	2017–2021
Age	45 (6–93)	46 (7–83)
**Menstrual status**
Premenstrual	4% (2)	4.5% (1)
Menstruating	49% (24)	50% (11)
post-menstrual/hysterectomy	47% (23)	45% (10)
**Sexual activity**
Yes	9% (5)	36% (8)
No	17% (9)	36% (8)
Not specified	75% (41)	32% (7)
**Diagnosis**
SJS	58% (31)	50% (11)
TEN	44% (22)	50% (11)
**Extent of vulvovaginal involvement**
Vulvar involvement	49% (26)	72% (16)
Vaginal involvement	40% (21)	41% (9)
Vaginal only	0	0
Vaginal involvement not specified	__	18% (4)
Gynecology consult	26% (14)	86% (19)
Dilator recommended (a)	38% (8)	59% (13)
Used	38% (8)	55% (12)
Steroid cream/ointment	38% (8)	55% (12)
No vulvar treatment recommendations +	31% (8)	6% (1)
Vulvar treatment recommendations (b)	69% (18)	100% (16)
Gauze only	19% (5)	0
Topical steroid	23% (6)	59% (13)
Other	23% (6)	56% (9)
Menstrual suppression (c)	38% (9)	73% (8)
Antifungal	4% (2)	50% (11)

*(a) recommendation for vaginal dilator of the patients with documented vaginal involvement (n = 21 from 2008 to 2017, n = 9 from 2017 to 2021). (b) Vulvar treatment recommendations of the patients with vulvar involvement (n = 26 from 2008 to 2017, n = 16 from 2017 to 2021). (c) menstrual suppression of menstruating patients (n = 24, n = 11)*.

## Discussion

SJS/TEN is a severe mucocutaneous reaction characterized by epidermal necrosis and mucosal sloughing. The extent of mucocutaneous involvement is variable and widespread, often affecting genitourinary tracts.

In our study, from 2008 to 2021, vulvovaginal involvement was documented overall in ~55% of SJS/TEN cases, compared to vulvovaginal involvement of up to 70% cited in the literature. Notably, when examining the patient cohorts pre and post clinical pathway implementation, the finding of vulvovaginal involvement increased from 49 to 72%. Gynecology consults also increased from 26% pre implementation to 86% post implementation. The increase in rates of vulvovaginal involvement in patients with SJS/TEN after the implementation of the clinical pathway likely represents an increase in the number of thorough pelvic evaluations, rather than an actual increase in the prevalence of the disease. Interestingly, we found no difference in the presence of vulvovaginal involvement between patients with SJS vs. TEN. Collectively these findings underscore the importance of having protocols in place with set treatment recommendations for primary teams to follow. While the severity of an individual case may not make the assessment and treatment of vulvovaginal lesions a priority, many patients with SJS/TEN will have genital involvement, and our findings suggest that severity of the disease does not correlate with the presence or absence of genital involvement and should not be a consideration in the decision of whether to do a pelvic exam.

We found a significant improvement in the percentage of patients who were treated for vulvovaginal SJS/TEN. While only 38% of patients received treatment with vaginal dilators pre-protocol, 59% received it after protocol implementation. Menstrual suppression and antifungals were also recommended more consistently following the implementation of the clinical pathway.

We were not able to gather information about the long-term outcomes of vulvovaginal involvement in the patients hospitalized prior to 2017, as the follow up was particularly low. There is still little information on how effective these treatments are in preventing adverse long-term outcomes.

With regards to future directions, we identified the need to improve clinical follow-up after discharge from the hospital which could be arranged in multidisciplinary visits and would allow the assessment of long-term symptomatic outcomes (pain, sexual activity, etc.). Of particular importance is the management of long-term sequelae like vaginal dryness, pain, dyspareunia. The role of local estrogen and vaginal laser as therapeutics still needs to be explored. Pelvic floor physical therapy might also play a key role in long-term rehabilitation. The possibility of experimental amniotic membrane application to affected vaginal walls, similar to the procedure in Ophthalmology, is promising and requires additional investigation. While our review demonstrates significant progress in the last 4 years, and highlights the importance of a clinical protocol, there is still a need for optimization of prevention and treatment of urogenital sequelae of SJS/TEN. We recommend a gynecology consult on all SJS/TEN patients seen by other services for a comprehensive assessment of genital involvement.

## Data Availability Statement

The raw data supporting the conclusions of this article will be made available by the authors, without undue reservation.

## Ethics Statement

The studies involving human participants were reviewed and approved by University of Washington IRB approval. Written informed consent from the participants or their legal guardian/next of kin was not required to participate in this study in accordance with the national legislation and the institutional requirements.

## Author Contributions

EF, MD, and SH have contributed with data collection, data evaluation, and writing and revising of the manuscript. All authors contributed to the article and approved the submitted version.

## Conflict of Interest

The authors declare that the research was conducted in the absence of any commercial or financial relationships that could be construed as a potential conflict of interest.

## Publisher's Note

All claims expressed in this article are solely those of the authors and do not necessarily represent those of their affiliated organizations, or those of the publisher, the editors and the reviewers. Any product that may be evaluated in this article, or claim that may be made by its manufacturer, is not guaranteed or endorsed by the publisher.
